# Differential Expression of *Sox11* and *Bdnf* mRNA Isoforms in the Injured and Regenerating Nervous Systems

**DOI:** 10.3389/fnmol.2017.00354

**Published:** 2017-11-02

**Authors:** Felix L. Struebing, Jiaxing Wang, Ying Li, Rebecca King, Olivia C. Mistretta, Arthur W. English, Eldon E. Geisert

**Affiliations:** ^1^Department of Ophthalmology, Emory University, Atlanta, GA, United States; ^2^Department of Ophthalmology, Tianjin Medical University General Hospital, Tianjin, China; ^3^Department of Cell Biology, Emory University, Atlanta, GA, United States

**Keywords:** axon injury, axon regeneration, gene expression, retinal ganglion cells, DRG neurons, glaucoma, epigenetic regulation, untranslated regions

## Abstract

In both the central nervous system (CNS) and the peripheral nervous system (PNS), axonal injury induces changes in neuronal gene expression. In the PNS, a relatively well-characterized alteration in transcriptional activation is known to promote axonal regeneration. This transcriptional cascade includes the neurotrophin *Bdnf* and the transcription factor *Sox11*. Although both molecules act to facilitate successful axon regeneration in the PNS, this process does not occur in the CNS. The present study examines the differential expression of *Sox11* and *Bdnf* mRNA isoforms in the PNS and CNS using three experimental paradigms at different time points: (i) the acutely injured CNS (retina after optic nerve crush) and PNS (dorsal root ganglion after sciatic nerve crush), (ii) a CNS regeneration model (retina after optic nerve crush and induced regeneration); and (iii) the retina during a chronic form of central neurodegeneration (the DBA/2J glaucoma model). We find an initial increase of *Sox11* in both PNS and CNS after injury; however, the expression of *Bdnf* isoforms is higher in the PNS relative to the CNS. Sustained upregulation of *Sox11* is seen in the injured retina following regeneration treatment, while the expression of two *Bdnf* mRNA isoforms is suppressed. Furthermore, two isoforms of *Sox11* with different 3′UTR lengths are present in the retina, and the long isoform is specifically upregulated in later stages of glaucoma. These results provide insight into the molecular cascades active during axonal injury and regeneration in mammalian neurons.

## Introduction

It is well known that neurons of the peripheral nervous system (PNS) have the ability to regrow damaged axons, while neurons of the central nervous system (CNS) often die following axonal injury (Young, 2014). This difference in regenerative capacity is partly attributed to the non-permissive growth environment of the CNS, with subsequent failure of neurons to re-myelinate their injured axons (Geisert et al., 1998; Yiu and He, 2006; Fernandes et al., 2016). The regeneration environment of the PNS is considered to be more permissive to axon regeneration. However, environment does not seem to be the only factor affecting regeneration differentially (Brosius Lutz and Barres, 2014). Neuron-specific transcriptional cascades are involved in promoting regeneration in the PNS and in the abrogative response in the CNS. The specific differences in these transcription cascades are not fully defined (Venkatesh and Blackmore, 2017). Among the genes implicated in the differential capacity for axon regeneration is brain-derived neurotrophic factor (BDNF), known to play a prominent role in the developing and injured PNS (Richner et al., 2014). BDNF is not only secreted from muscles, but also from Schwann cells in the PNS and regenerating axons in the CNS. It can bind to axonal tropomyosin receptor kinase B (trkB) receptors, resulting in axon elongation (Segal et al., 1995), dendritic or synaptic growth, or neurogenesis (Lu et al., 2014). In line with a role for BDNF/trkB signaling in axon regeneration, application of small molecules acting as agonists on the trkB receptor were shown to increase axon growth after PNS injury, independent of endogenous BDNF expression (English et al., 2013). Inhibiting *Bdnf* expression in Schwann cells abolished axon regeneration, but the phenotype could be rescued by electrical stimulation or exercise. This rescue was the result of increased secretion of BDNF from neurons, suggesting that sustained neural activity can also lead to increased neuronal *Bdnf* expression that acts in an autocrine manner to promote axon regeneration (Wilhelm et al., 2012). Interestingly, *Bdnf* signaling is known to affect motor and sensory nerves differentially, as it was shown that mice lacking *Bdnf* expression developed sensory and not motor deficits (Ernfors et al., 1994), but high doses of exogenous BDNF could selectively promote motor axon outgrowth after sciatic nerve transection (Santos et al., 2016). Therefore, the molecular networks up- and downstream of the BDNF/trkB signaling pathway can partly mediate the regenerative response in neurons in a cell type-specific way.

One of the genes known to drive *Bdnf* expression is *Sox11*. This transcription factor is expressed in neuronal precursors and retinal ganglion cells (RGCs), making it indispensable for neuron differentiation (Mu et al., 2012; Salerno et al., 2012). Overexpression of *Sox11* after peripheral nerve injury resulted in enhanced regeneration and improvement of measurements of functional recovery (Jankowski et al., 2009; Jing et al., 2012). Conversely, overexpression of *Sox11* after spinal cord injury lead to impaired motor dexterity despite an enhanced regenerative capability of CNS axons (Wang et al., 2015). In the mouse retina, *Sox11* can activate transcripts associated with axon growth, while suppressing some genes involved in synapse formation (Norsworthy et al., 2017). *Sox11* also differentially affects survival and regeneration of distinct RGC subtypes and is a downstream effector of Dual Leucine Zipper Kinase (DLK/*Map3k12*) following axon injury (Welsbie et al., 2017). Furthermore, its overexpression results in substantial axonal regeneration (Norsworthy et al., 2017). These data suggest that *Sox11* acts as a regulatory switch between cell survival and axonal growth and that it selectively exploits a tissue- as well as cell-specific molecular environment to modulate gene expression and overall cell function.

Among other (epi)genetic mechanisms, this kind of specificity can be conferred by different mRNA isoforms. For example, it is now well established that in the mouse *Bdnf* gene, eight 5′ non-coding exons are alternatively spliced to create nine distinct mRNA isoforms (Aid et al., 2007). All of these different mRNAs include the same 3′ coding exon (exon IX), and thus encode the same protein, but they differ in regard to their subcellular location (Bishop et al., 1994; Aid et al., 2007). While no similar mRNA isoforms of *Sox11* have been described yet, this single-exon gene underwent a remarkable reannotation since its first discovery (Wright et al., 1993) primarily due to differences in 3′Untranslated Region (3′UTR) length. It is currently annotated with a 3′UTR length of almost 7 kb. Whereas this would be considered unusually long for a non-neural cell, 3′UTRs are frequently elongated in neurons (Miura et al., 2014). It was also shown that differences in UTR length can have functional effects. For example, expression of the same gene with different UTR lengths was recently observed in different cellular compartments (Berkovits and Mayr, 2015), implicating extended 3′UTRs in an RNA-binding protein mediated, post-transcriptional regulatory process. Similarly, expression of *Sox11* coding (exon) and non-coding (3′UTR) segments was found to be spatially separated in mouse neuronal tissue (Kocabas et al., 2015). Expression of its protein-coding region (CDS) was higher in hippocampal strati that underwent active neurogenesis, whereas expression of its 3′UTR was restricted to terminally differentiated cells. Therefore, the 3′UTR-to-CDS ratio of certain genes confers a distinct and tissue-specific regulatory mechanism.

Both *Bdnf* and *Sox11* are genes that play important roles in axon regeneration. Transcription of each gene is regulated in multiple ways. Thus, we began a series of experiments to compare and contrast the response of these genes under different conditions. The present study examines the transcriptional response of *Sox11* and *Bdnf* in the PNS and CNS in regenerating and non-regenerating paradigms.

## Materials and Methods

### Animals

For each experimental group and time point, an equal number of male and female C57BL/6J mice (*n* ≥ 4 per group) underwent the procedures described below (except for sciatic nerve crush where *m* = 10 and *f* = 11). Power analysis for this sample size and three pairwise comparisons demonstrated a 96% chance of detecting a true twofold change with a standard deviation of 25% and a Type I error rate of 5%. All C57BL/6J animals were between 60 and 100 days of age. Mice were maintained on a 12 h light – 12 h dark cycle in a parasite-free facility with food and water *ad libitum*. All procedures involving animals were approved by the Animal Care and Use Committee of Emory University and were in accordance with the ARVO Statement for the Use of Animals in Ophthalmic and Vision Research.

### Optic Nerve Crush

Optic nerve crush was performed as described in Templeton and Geisert (2012). Briefly, C57BL/6J mice were anesthetized using Ketamine (100 mg/kg) and Xylazine (15 mg/kg). Under the binocular operating scope, a small incision was made in the conjunctiva. With micro-forceps (Dumont #5/45 Forceps, Roboz, cat. #RS-5005, Gaithersburg, MD, United States), the edge of the conjunctiva was grasped next to the globe. The globe was rotated nasally to allow visualization of its posterior aspect and optic nerve. The exposed optic nerve was then clamped 2 mm distal from the optic nerve head with Dumont #N7 self-closing forceps (Roboz, cat. #RS-5027) for 10 s. At the end of the procedure, a drop of 0.5% proparacaine hydrochloride ophthalmic solution (Falcon Pharmaceuticals, Fort Worth TX, United States) was administered for pain control and a small amount of surgical lubricant (KY Jelly, McNeil-PPC, Skillman, NJ, United States) was applied to the eye to protect it from drying. Mice were allowed to recover on a heating pad while being monitored until fully awake.

### Sciatic Nerve Crush

Ten male and 11 female mice were anesthetized with isoflurane (1%) and the sciatic nerve was exposed in the posterior mid-thigh. The nerve was then crushed midway between the sciatic foramen and the branching into common fibular, tibial, and sural nerves, using the same forceps used to crush optic nerves. Pressure on the forceps was held for 10 s. When pressure was released, a clear space in the nerve at the crush site, indicating an effective crush, could be observed in all cases. Surgical wounds were closed in layers and animals received a single dose of Meloxicam (5 mg/kg, po). Mice were allowed to recover on a heating pad while being monitored until fully awake. All procedures were performed bilaterally.

### Regeneration Treatment and Vectors

Two weeks prior to optic nerve crush, mice were injected intravitreally with 2 μL of *Pten*-shRNA-GFP packaged into AAV2 backbone constructs (titer = 1.5^∗^10^12^ vg/ml). The shRNA target sequence was previously validated and is described in (Zukor et al., 2013). PTEN knockdown was verified by immunostaining an AAV-transduced retina with a primary antibody against PTEN (Cell Signaling Technology Rabbit mAb 138G6) and a secondary antibody as described previously (Struebing et al., 2017). Intravitreal injection of an Alexa Fluor 647-conjugated anterograde neurite tracer Cholera toxin B (Invitrogen C34778) 2 days prior to sacrifice demonstrated successful axon regeneration past the optic nerve crush site using this model (**Supplementary Figure S3**). Fundus fluorescence (GFP) was monitored for successful retinal transfection on a Bioptigen SD-OCT. Mice without GFP fundus signal were excluded from the study. Two weeks after the AAV injection, eyes were injected with a mix of Zymosan and 8-CPT-cAMP (Sigma) (total volume 2 μL) which was immediately followed by ONC as described above. Co-delivery of Zymosan/8-CPT-cAMP and *Pten*-shRNA was previously shown to augment optic nerve regeneration more than 10-fold by induction of a low-grade inflammatory state (Kurimoto et al., 2010). GFP for both AAV-GFP and AAV-Pten-shRNA was under control of the CAG promoter. Both plasmids used the same pAAV backbone and AAV-GFP was titered to 1.2^∗^10^13^ vg/ml.

### DBA/2J Glaucoma Model

To study the effects of glaucoma, female DBA/2J mice (*n* = 36) were sacrificed between 280 and 320 days of age. The retina was quickly separated from the optic nerve and placed in RNA-inhibitor containing buffer as described below. Care was taken not to exert any force on the optic nerve, which was post-fixed in 2% Paraformaldehyde and 2% Glutaraldehyde in Phosphate Buffer. The optic nerve was then osmicated and embedded in plastic. Semi-thin (0.7 μm) sections were cut and stained with 1% p-phenylenediamine (Sigma) for 30 min. Optic nerve photographs were taken with an Olympus BX-51 microscope at 20× magnification and graded by two blinded reviewers according to the degree of damaged axons present in sections. PCR reactions for DBA/2J glaucoma samples were run individually, and the investigator was blinded to the optic nerve damage during analysis. Groups were then clustered by optic nerve damage after data normalization.

### RNA Isolation

For each experimental time point, mice were deeply anesthetized with Ketamine/Xylazine as described above and sacrificed by rapid cervical dislocation. Retinas or L4 dorsal root ganglia were quickly dissected under a dissection microscope and directly placed into 160 U/ml Ribolock^®^ (Thermo Scientific, Walton, MA, United States) in Hank’s Balanced Salt Solution (Sigma, St. Louis, MO, United States) on ice. Tissue was stored at -80°C. RNA was isolated in batches using a Qiacube and the RNeasy Mini Kit (Qiagen, Hilden, Germany) according to the manufacturer’s instructions. The isolation included on-column DNase1 treatment to remove contaminating genomic DNA. All tissue was harvested between 10 am and noon to minimize circadian differences in gene expression. RNA integrity was assessed on a Bioanalyzer 2100 (Agilent, Santa Clara, CA, United States). Samples with an RNA integrity score (RIN-score) less than 8.0 were not used in the study. For three DRG samples, a RIN-score could not be determined due to sub-threshold RNA concentrations. For these samples, RNA quality was assessed by 28S-18S rRNA gradient and only samples with a ratio ∼2 were used. RNA was then quantified by spectrophotometry and 260/280 ratios for all samples were >2.1.

### Reverse Transcription

First strand synthesis was carried out using PrimeScript RT Kit (Takara Bio, Shiga, Japan). For each sample, 300 ng of total RNA were reverse transcribed following the manufacturer’s instructions. To further decrease genomic DNA contamination, RNA was incubated for 5 min in gDNA eraser (Takara) at 42°C and then immediately cooled on ice. Reverse transcription took place at 42°C for 20 min and a mix of random hexamers and oligo-(d)T primers was used to prime the reactions. cDNA was diluted 100-fold to a final concentration of 0.3 ng/μL RNA equivalent with ultrapure H2O and stored at 4°C.

### Primer Design and Validation

Primers for *Sox11* were designed using NCBI Primer Blast with targeted annealing temperature of 61–64°C after correction for 3.5 mM Mg^2+^. For *Bdnf*, we used the primers previously validated and published in (Salerno et al., 2012). No *in silico* off-targets were found by BLASTing the primer sequences. All primers were checked for specificity by melting curve analysis and Sanger sequencing of amplicons. Primer sequences are given in **Supplementary Table S1**. All primers were evaluated in digital PCR reactions for linear amplification efficiency and a clear separation between negative and positive fluorescent droplets. *Ppia* was used as a reference gene (Quantitect Primer Assay, Qiagen). We chose *Ppia* because (i) its expression level in DRG and retina is within the dynamic range of ddPCR and (ii) because its expression is very stable after crush procedure (**Supplementary Figure S4**). Additionally, this reference gene is used throughout the literature for qPCR of neuronal cells (He et al., 2015).

### Digital Droplet PCR

A total of 1,038 20 μL reactions were distributed onto 96-well-plates using QX200 ddPCR EvaGreen Supermix (Bio-Rad, Hercules, CA, United States) according to the manufacturer’s instructions. The final primer concentration was 200 nM and 5 μL of cDNA were used for each reaction. Droplets were generated automatically on a QX200 Droplet Generator (Bio-Rad). PCR was carried out on a C1000 Touch Thermal Cycler (Bio-Rad) with the following parameters: Initial activation at 95°C for 5 min, followed by 40 cycles of denaturation (95°C, 30 s) and combined annealing/elongation (60°C, 60 s) and a ramp rate of 2°C/sec. The droplet signal was stabilized for 5 min at 4°C followed by 5 min at 90°C according to the QX200 ddPCR EvaGreen Supermix protocol. Droplets were then read with a QX200 Droplet Reader (Bio-Rad).

### Digital Droplet PCR Analysis

Absolute values of ddPCR products (copies/μL) including 95% confidence interval were calculated by QuantaSoft software (Bio-Rad) by fitting the fraction of positive droplets to a Poisson distribution (Gutierrez-Aguirre et al., 2015). The fluorescence threshold was adjusted manually and kept constant for each reaction that used the same primer to avoid batch effects. Normalization to *Ppia* was carried out by first calculating the average *Ppia* concentration (*C*_p_) across all samples and then multiplying each sample concentration *C*_i_ by a calibrator M = $M=\frac{C_{i}}{\left(C_{p}\right)}$. Absolute levels were transformed to log_2_-based fold-changes for plotting purposes. Genomic DNA (gDNA) contamination was assessed with a primer pair (mVPA) designed to amplify only non-expressed genomic regions (Laurell et al., 2012). There were 11.4 copies of mVPA in 0.3 ng/μL gDNA, while all cDNA samples had mVPA concentrations < 0.25 copies for the same concentration throughout (more than 50% of the samples were completely free of gDNA according to this method). Thus, there was negligible gDNA contamination, contributing less than 2.5% to the total fluorescence signal after thermal cycling. Analysis of variance followed by Tukey’s HSD *post hoc* test was used for statistical testing.

### Rapid Amplification of cDNA Ends (RACE)

One μg of total RNA from normal C57BL/6J retinas and such that underwent ONC (*n* = 6 each) was isolated as described above and used for RACE experiments. 5′RACE was performed according to the manufacturers protocol (Ambion First Choice RLM-RACE Kit, #AM1700). For 3′ RACE, the protocol was adapted as follows: RNA was mixed with 150 nM custom 3′RACE adapter (DNA primer: 5′-CCTATAGTGAGTCGTATTAATTCTGTGCTCGC-3′) and 15 units of T4 RNA ligase 2 (New England Biolabs, M0239) in ligase buffer. Incubation for 1 h at 37°C resulted in ligation of free RNA 3′OH ends to the 5′ ends of the adapter primer. A reverse-complement 3′ RACE RT adapter (5′-GCGAGCACAGAATTAATACGACTCACTATAGG-3′ was then added and the reaction was heated for 5 min to 65°C to allow annealing. This was followed by random hexamer-dependent reverse transcription with Superscript IV RT enzyme (Invitrogen) according to the manufacturer’s instructions and including an RNAse H digestion step at the end. Nested PCR was then carried out with a mix of gene-specific primers (**Supplementary Table S1**) and 3′RACE outer (5′-GCGAGCACAGAATTAATACGACT-3′), followed by 3′RACE inner primer (5′-CGCGGATCCGAATTAATACGACTCACTATAGG) using AccuPrime High Fidelity Polymerase (Invitrogen) and the following cycling conditions: 94°C initial denaturation for 30 s, and 35 cycles of 20 s at 94°C, 30 s at 65°C, 8 min at 68°C. Amplicons were then separated by agarose gel electrophoresis and products were purified (NucleoSpin Gel and PCR Clean-up kit, Macherey-Nagel, Düren, Germany, #740609.10) for Sanger sequencing.

### Data Sources and Bioinformatics

ChIP-Seq data were downloaded from the NCBI sequence read archive, mapped to 10 mm using bowtie2 and converted to genome coverage-normalized bigwig graphs with deeptools. Biological replicates were merged prior to conversion. All datasets were retina-specific and created using C57BL/6 mice. The following SRA accession IDs were used: SRX1365314, SRX1365315, SRX1365318, SRX1365319, SRX1365306, SRX1365307, SRX1365313, SRX1365312, SRX1365324, SRX1365323, SRX1365329, SRX1365330 (Aldiri et al., 2015). CAGE data was downloaded from the FANTOM 5 consortium in tab-delimited format mapped to mm9 (Lizio et al., 2015).

## Results

### Analysis of the *Sox11* Locus

**FIGURE 1:**
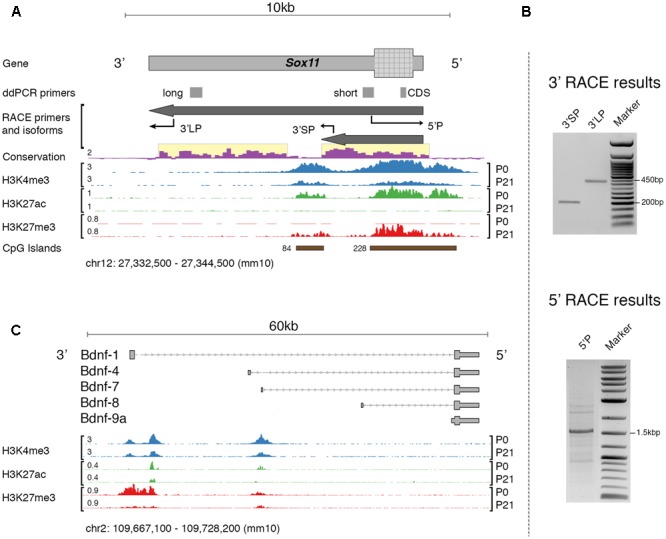
**(A)** Overview of the mouse *Sox11* locus. The locations of primers for rapid amplification of cDNA ends (RACE) and gene expression testing (ddPCR primers) are shown together with the identified isoforms in the retina. Conservation score is given for multiple alignments of vertebrate genomes (conservation scoring by phylogenetic *p*-values from the PHAST package for multiple alignments of 59 vertebrate genomes to the mouse genome; phyloP60). Below, retina-specific ChIP-Seq coverage for three histone modifications is shown at P0 and P21, respectively. The number in ‘CpG Islands’ represents the max. number of consecutive CpG base pairs. Note the highly conserved 3′UTRs for both isoforms (highlighted in yellow). The histone features and concurrent CpG islands suggest the existence of two promoter-like states, one over the *Sox11* 5′UTR and CDS, and one within its 3′UTR. There is a switch from active (H3K27ac) to silenced (H3k27me3) chromatin between P0 and P21, and it appears that H3K27 trimethylation is increased over *Sox11* CDS compared to its 3′UTR. Similar data does not exist yet for adult mouse retina. **(B)** Agarose gel electrophoresis of RACE products using the primers described in **(A)**. Bands were purified and sequenced. Specificity was confirmed by aligning sequence results to the *Sox11* locus. Only the major band at ∼1.5 kb in the 5′RACE gel, but both bands in the 3′RACE gel resulted in specific products. **(C)** Overview of the mouse *Bdnf* locus and isoforms targeted during expression testing. The histone profile for this locus is shown for comparative reasons. Note the presence of 2–3 active promoters as indicated by positive H3K4me3 signal and the preferential silencing (H3K27me3) of the proximal promoter at P0.

The primary interest of the present study is the expression of specific mRNA isoforms of *Sox11* and *Bdnf*. In the mouse, the expression of specific mRNA isoforms of *Bdnf* is relatively well defined (Aid et al., 2007). This is not the case for *Sox11*. Therefore, the first step in our analysis was to re-evaluate the *Sox11* gene locus in adult mouse and the expression of isoforms in retinal tissue (**Figure 1A**). An earlier study using serial analysis of gene expression found evidence for alternative polyadenylation sites as well as antisense transcripts originating from this locus during mouse corticogenesis (Ling et al., 2009). We performed 5′- and 3′- rapid amplification of cDNA ends (RACE) assays in adult mice in an optic nerve crush (ONC) and a normal condition to specify transcription start (TSS) and end site, respectively. Because of the presence of at least 9 Poly-A stretches (defined as >8 consecutive A) within the main *Sox11* transcript, 3′RACE with the standard oligo(d)T adapter primer resulted in false-positive 3′ tails terminating at one of these sites (Nam et al., 2002). We therefore modified the protocol so that it was independent of oligo(d)T priming. Using this method, we identified specific bands consistent with two different 3′UTR lengths that both aligned to the *Sox11* locus (verified by Sanger sequencing, **Figure 1B**). We observed no difference in mRNA isoforms between the ONC and the control situation, which was additionally validated by visually inspecting genome graphs of a published ONC RNA-seq dataset (Yasuda et al., 2014). Interestingly, the short *Sox11* isoform terminated just upstream of an intragenic CpG island that was only marginally conserved across species (**Figure 1A**). In comparison, the TSS-associated CpG-island was relatively conserved. Investigating the epigenomic profile of this locus using publically available data (Aldiri et al., 2015) suggested that both CpG islands possessed features reminiscent of developmentally regulated promoters. First, both were enriched in the promoter-associated histone mark H3K4me3 (Shen et al., 2012). While to a lesser degree, this mark was still present in adults. Second, the switch from Histone H3 Lysine 27 acetylation to trimethylation (H3K27ac → H2K27me3) between P0 and P21 likely represented gene silencing (Tie et al., 2016). This was consistent with the time course of *Sox11* expression during development, as the expression of this gene decreased rapidly after birth (Ling et al., 2009).

We then could confirm the previously established, canonical 5′ start site on the minus strand using cap-dependent 5′RACE. Other TSSs were not found and there was no difference between ONC and control; however, exploration of cap analysis of gene expression data (CAGE-seq) provided by the FANTOM consortium, a sequencing method that can detect TSSs (Hon et al., 2017), revealed intragenic sense and antisense transcription start sites at different development stages (**Supplementary Figure S1**). At the same time, CAGE data also confirmed the sole canonical 5′TSS in adult. It should be noted that we were not able to reliably amplify any message from the antisense strand. Based on these results, we suggest that *Sox11* in the adult retina is present in a long (7,812 nt) and a short (2,842 nt) isoform with identical TSSs but different 3′UTR lengths. Furthermore, the histone profile indicates that *Sox11* is epigenetically silenced in adults but may be reactivated when needed, as the promoter retains its active signature mark.

### Expression of *Sox11* and *Bdnf* in the Injured CNS and PNS

**FIGURE 2:**
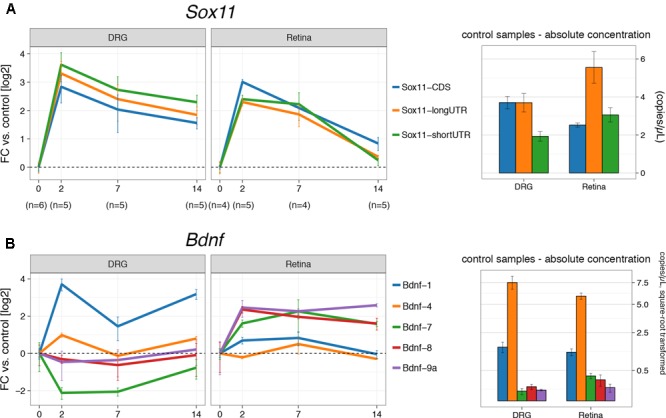
Digital droplet PCR gene expression results for crush procedures. **(A)** The left part shows fold changes (log_2_) for DRG and retina for the three *Sox11* primers used at 2, 7, and 14 days after the respective crush procedure. The control group (0 days) received a sham procedure. Absolute concentration in mRNA copies/μL for control samples (‘starting amount’) is given on the right-hand side. There is an overabundance of the *Sox11* long 3′UTR isoform in the retina, while the short 3′UTR is underrepresented in DRG (*p* < 0.05). Nevertheless, the fold-changes in both tissues suggest co-regulation of all transcripts. Notice the faster decay of *Sox11* in the retina compared to DRG. **(B)** Equivalent to **(A)**, but for *Bdnf* isoforms 1, 4, 7, 8, and 9a, among which *Bdnf-4* is the most abundant isoform in Retina and DRG, followed by *Bdnf-1*. Other isoforms are barely expressed. The y-scale on the right hand-side of the picture was square-root transformed to better show differences at lower concentrations. Error bars for fold-changes represent standard error, while the 95% CI is given for absolute concentrations.

To examine the role of the two *Sox11* isoforms in axon regenerating and non-regenerating scenarios, the temporal expression patterns of *Sox11* were defined after either ONC or sciatic nerve crush (SNC). Samples from the retina and the L4 dorsal root ganglion were taken at 2, 7, and 14 days after injury. For each control group at 0 days, we performed a sham surgery (identical anesthesia + surgical cuts less the crush). Each of the mRNA isoforms was targeted using primers specific for the short and long version of *Sox11* 3′UTR. In addition, primers were used to test whether or not differences in expression of the protein-coding region itself were found (**Figure 1A**). We observed almost equal upregulation (∼8-fold) of all *Sox11* isoforms between 0 and 2 days (*p* < 0.01) after crush in both tissues (**Figure 2A**). While mRNA levels remained elevated ∼4-fold at 14 days in DRGs, they returned to just above baseline in the retina at this time. Even though approximately equal fold *changes* between isoforms were found in both tissues, their starting (pre-injury) amount differed. For example, the long 3′UTR was expressed almost three times higher than short 3′UTR and CDS in retina control samples. This observation was corroborated by retinal microarray data taken 2 and 5 days after ONC, hosted on GeneNetwork and created previously by our group (**Supplementary Figure S2**). In DRGs, only the long 3′UTR was significantly increased between 0 and 2 days (*p* = 0.03), but the short *Sox11* 3′UTR was only expressed at half the concentration of CDS and long 3′UTR. These data argue for a non-linear relationship of CDS to UTR, suggesting differential regulation, post-transcriptional separation or selective degradation of mRNA isoforms, possibly in different cellular subtypes.

The next step in the analysis was to test the expression of *Bdnf* mRNA isoforms after ONC and SNC (**Figure 2B**). Each of the *Bdnf* mRNA isoforms is created by splicing one of its eight 5′ non-coding exons to a common 3′ protein-coding exon. Our numbering system reflects which of those exons is a part of the isoform; e.g., *Bdnf*-4 is the mRNA containing 5′ exon IV. For *Bdnf*-9a, the mRNA isoform does not contain a spliced 5′ exon; transcription is thought to be initiated in the intron before the protein-coding exon. For the purpose of this project, we used primers for five of its nine exons, which were previously determined to be specifically regulated by *Sox11* (Salerno et al., 2012). In control samples, *Bdnf*-4 was the most prevalent isoform in retina and DRG, followed by *Bdnf*-1 (**Figure 2B**). The remaining isoforms studied (*Bdnf*-7, *Bdnf*-8, and *Bdnf*-9a) were barely expressed in DRG and only slightly higher in retina. In DRG, there was a strong (>10-fold) increase of *Bdnf*-1 at 2 days (*p* < 0.001) and 14 days (*p* < 0.001) over the control situation, with a significant transient drop from 2 to 7 days (*p* < 0.001). A similar pattern was found for *Bdnf*-4 (*p* = 0.04, 0 days vs. 2 days, other comparisons n.s.), even though its expression only doubled. However, the opposite was true for *Bdnf*-4 expression in retina; here, *Bdnf*-4 expression was decreased at 2 and 14 days and increased at 7 days, even though this change did not reach statistical significance. Additionally, a transient upregulation of *Bdnf*-1 was found at 2 days (*p* < 0.001) and 7 days but dropped to baseline levels 14 days after ONC. Despite their relatively low absolute expression levels, other *Bdnf* isoforms were increased at all time points in the retina (*p* > 0.05), but either suppressed (*Bdnf*-7) or changed little in DRG. Thus, *Bdnf* isoform expression following axon injury varies decisively between DRG and retina.

### Regeneration Treatment of the Injured Retina Influences *Bdnf* and *Sox11* Expression

**FIGURE 3:**
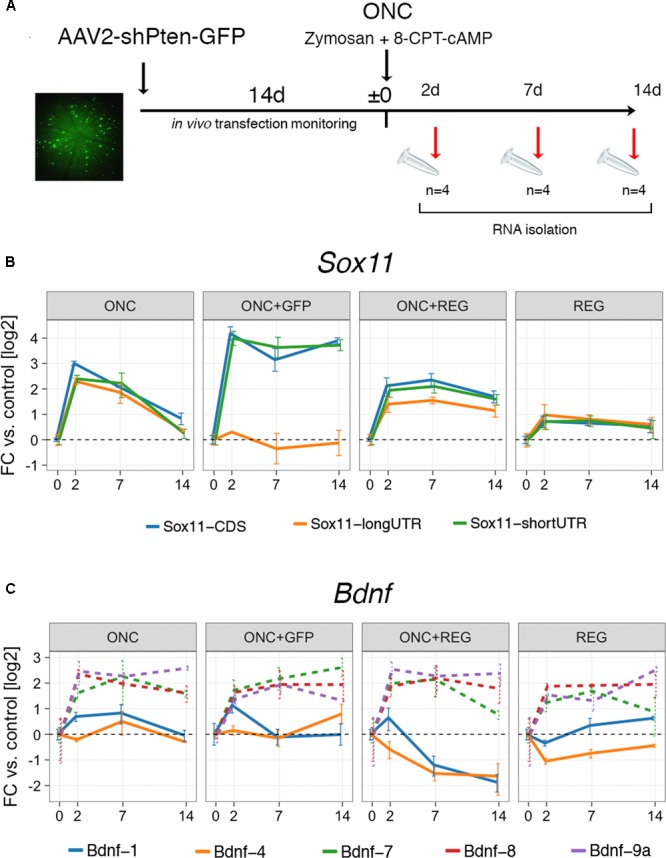
Digital droplet PCR gene expression results for ONC after regeneration treatment. **(A)** Timeline of the experimental interventions for the regeneration protocol. *Pten* expression was silenced using shRNA delivered by GFP-tagged AAV via intravitreal injection and compared to an ONC + AAV-GFP control vector (ONC+GFP) and a regeneration treatment only (REG) group. After 2 weeks, eyes were injected with Zymosan-cAMP mix and subjected to ONC. Samples were collected at 2, 7, and 14 days. The fundus image on the left is an example of one of the animals used in the study. Bright green cells are successfully transfected retinal ganglion cells. **(B)** Gene expression results for *Sox11* in log2-transformed fold changes. The groups labeled “REG” and “ONC+REG” received the regeneration treatment (AAV-*Pten*-shRNA-GFP + Zymosan/cAMP analog mix). REG slightly increases global *Sox11* expression, which is exacerbated by additionally subjecting mice to ONC (ONC+REG). Treatment with the AAV-GFP control vector and ONC (ONC+GFP) result in dissociated expression changes for *Sox11* short 3′UTR and long 3′UTR isoforms (*p* = 0.005 at 14 days). **(C)** Same as in **(B)**, but for *Bdnf* isoforms. While there is little change in the prevalent isoforms (1 and 4) in the REG only group, REG plus ONC lead to a prolonged suppression in expression. *Bdnf* minor isoforms show little differences between conditions (all changes n.s. with *p* > 0.05, dashed lines). Error bars represent standard error.

The clear and distinct differences in the expression of *Sox11* and *Bdnf* in neurons whose axons regenerate well in the PNS and those that do not regenerate well in the CNS prompted us to look at an experimental scenario in which CNS regeneration was enhanced (**Figure 3**). A GFP-tagged AAV vector containing short hairpin RNA (shRNA) targeting *Pten* was injected into the vitreous of the left eye. During the following 2 weeks, successful transfection was confirmed by monitoring fundus GFP fluorescence *in vivo* using an SD-OCT machine. After 2 weeks, ONC was performed, directly followed by injection of a Zymosan/cAMP analog mix, which is known to induce low-grade inflammation in the retina (Kurimoto et al., 2010). Tissue was harvested either 2, 7, or 14 days thereafter. Immunostaining for PTEN in a retinal flat mount demonstrated complete loss of PTEN expression in GFP-positive cells, and injection of an anterograde neurite tracer 2 days prior to sacrifice indicated strongly enhanced growth of retinal ganglion cell (RGC) axons past the crush site, verifying the efficacy of our approach (**Supplementary Figure S3**). We additionally performed two control experiments: One ONC experiment where only GFP and not *Pten* shRNA was delivered via AAV vectors (“ONC+GFP,” **Figure 3**), and one where only the regeneration treatment was provided and the nerve was not crushed to assess the effect of regeneration treatment alone on gene expression (“REG,” **Figure 3**). While REG resulted in only mild upregulation of *Sox11* isoforms, combined ONC+REG caused a >4-fold increase for the short *Sox11* isoform and CDS (*p* < 0.02) and a >2-fold increase for the long 3′UTR isoform (*p* = 0.012, **Figure 3B**). This increase also appeared to decay more slowly than in mice with ONC only (**Figure 2A**). We found an even stronger, almost 16-fold persistent upregulation of the *Sox11* CDS including its short 3′UTR in ONC eyes treated with the AAV-GFP control vector (*p* < 0.001), yet the expression levels of the long 3′UTR did not change significantly at any time under this situation (*p* > 0.9). While we expected to see no differences in gene expression between the ONC and ONC+GFP group, these results strongly argue for a dissociated regulation of *Sox11* short and long 3′UTR isoforms. They also further support the existing notion that either AAV, GFP or the combination of both can have unanticipated consequences on gene expression (Ansari et al., 2016; Berns and Muzyczka, 2017).

In contrast to *Sox11*, we did not find any differences in the expression of *Bdnf* isoforms between the ONC and the ONC+GFP groups. REG alone caused virtually no change in *Bdnf*-1 or *Bdnf*-4 expression; however, combined ONC+REG treatment resulted in prolonged suppression of the expression of both of these mRNA isoforms (*p* < 0.01 for both, **Figure 3C**). *Bdnf*-7, *Bdnf-*8 and *Bdnf*-9a all showed consistent fourfold upregulation, but these isoforms were very lowly expressed to begin with (**Figure 2B**, right panel) and hence the difference was not statistically significant.

In summary, these data demonstrate that the expression of *Sox11* isoforms is modulated by AAV administration and ONC to a higher degree than the regeneration treatment alone. While silencing *Pten* combined with induction of low-grade inflammation in the adult retina after injury results in synchronized upregulation of all *Sox11* isoforms and decreased expression of *Bdnf-1* and *Bdnf-4*, injection of an AAV-GFP control vector leads to stronger activation of *Sox11* CDS and short 3′UTR.

### Expression of *Sox11* and *Bdnf* during Chronic CNS Degeneration

**FIGURE 4:**
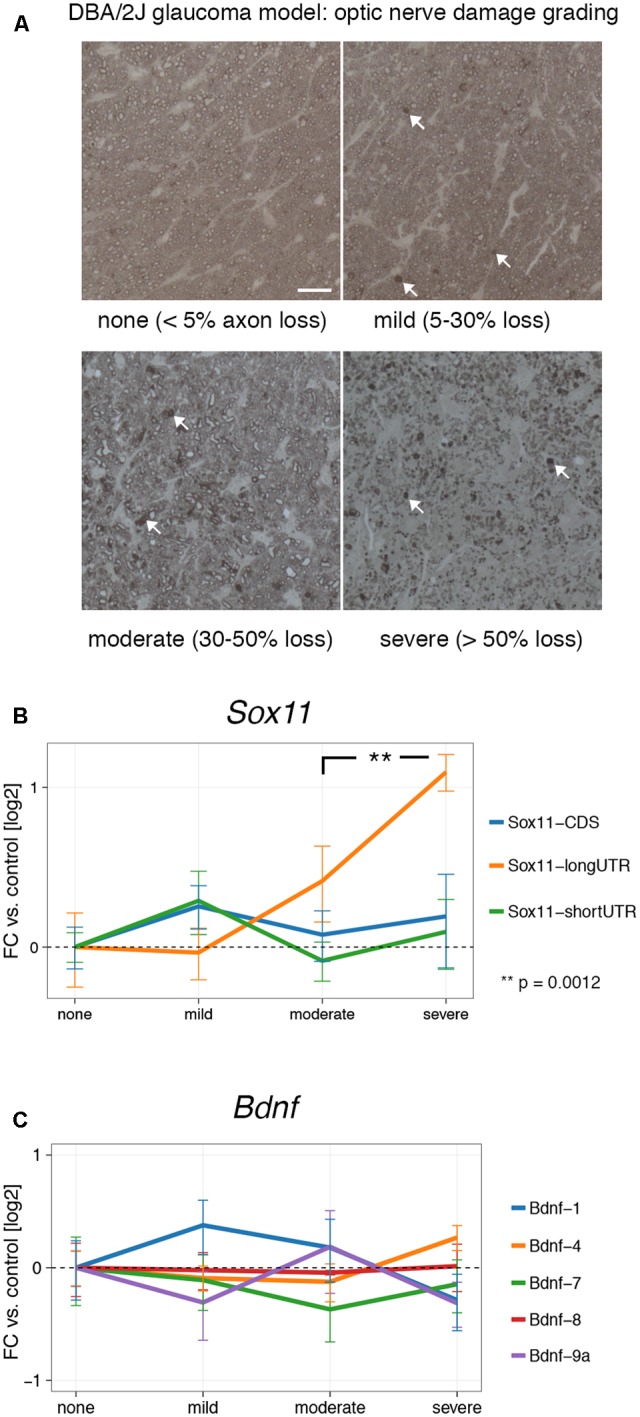
Expression of *Sox11* and *Bdnf* at different stages of glaucoma. **(A)** Samples were staged by optic nerve damage as seen in PPD-stained thin sections according to the percentage of degenerating axons shown in parentheses. Arrows point to accumulated myelin, which represents degenerating axons. Scale bar = 25 μm. **(B)** Only the *Sox11* long 3′UTR changes significantly with increasing optic nerve damage. All log_2_ fold changes were calculated in relation to the “none” glaucoma sample. **(C)** No significant changes exist for *Bdnf* at different glaucoma stages.

Our results show that *Sox11* and *Bdnf* are reactive to acute injury in the CNS and PNS, and that their expression is influenced by regenerative treatment prior to injury. However, gene expression can differ markedly between chronic and acute injuries. In order to test whether this was the case for *Sox11* and *Bdnf*, we examined their mRNA levels at different stages of glaucoma, the most common neurodegenerative disease (Jutley et al., 2017). To examine the effects of low-grade chronic injury, we used the DBA/2J mouse model of glaucoma, in which mutations of two genes (*Tyrp1* and *Gpnmb150*) cause iris pigment dispersion and subsequent elevation of intraocular pressure beginning at approximately 6 months of age (Anderson et al., 2002). This elevation in intraocular pressure results in retinal ganglion cell death and optic nerve degeneration (Howell et al., 2007). We isolated retinal RNA from 36 aged DBA/2J mice. The severity of glaucoma in each eye was defined by examination of the corresponding optic nerve. The optic nerve damage was stratified by the number of degenerating axons and expression of *Sox11* and *Bdnf* isoforms was examined in all samples (**Figure 4A**). Interestingly, there was a significant and steady increase in levels of *Sox11* long 3′UTR message in moderate up to more than twofold in severe glaucoma stages (**Figure 4B**) (*p* = 0.0012). In contrast, we did not find significant differences for *Bdnf* at any glaucoma stage (**Figure 4C**). These data demonstrate that *Sox11* isoforms are specifically regulated in a chronic neurodegenerative context.

## Discussion

The present study examined the expression of two genes, *Sox11* and *Bdnf*, that are known to play significant roles in the response of neurons to injury. Both have distinct expression patterns after axon damage and are directly involved in CNS and PNS regeneration. While peripheral and central neurons share about 90% of expressed genes, specific molecules are selectively expressed in both transcriptomes (Smith et al., 2011). This suggests that neurons in the CNS and PNS are not equal in their transcriptional program, which could explain their differing reactions to injury. Because the overexpression of either *Sox11* or *Bdnf* previously resulted in increased regenerative ability of CNS axons (Dawson et al., 2015; Wang et al., 2015; Santos et al., 2016; Norsworthy et al., 2017), we sought out to investigate their expression levels following crush injury in the regenerating PNS, the non-regenerating CNS, and the regeneration-stimulated CNS.

While *Sox11* was strongly up-regulated after both sciatic nerve and optic nerve crush, distinct tissue-specific differences existed. First, *Sox11* expression dynamics were prolonged in DRG neurons compared to retina. Because this transcription factor is not only associated with neuron differentiation but also axon growth and synapse formation (Jayaprakash et al., 2016), prolonged *Sox11* expression appears to be necessary to activate genes that are needed for successful axon regeneration. When we stimulated optic nerve regeneration by inhibiting *Pten* and induced mild inflammation, we observed sustained, fourfold higher *Sox11* expression in the retina. While this upregulation of retinal *Sox11* was also observed in the regeneration treatment control group not subjected to ONC, it was enhanced by the crush procedure. These findings are consistent with a functional role of *Sox11* in affecting transcription of regeneration-associated genes. In a recent study, overexpression of *Sox11* and simultaneous deletion of *Pten* led to slightly decreased RGC survival after ONC compared to *Pten* deletion alone, but also resulted in strongly increased axon regeneration throughout the entire length of the optic nerve (Norsworthy et al., 2017). In the same study, *Sox11* overexpression resulted in the death of alpha-RGCs, a subtype that would normally survive and regenerate preferentially following *Pten* knockdown (Duan et al., 2015). However, overexpression of *Sox11* strongly enhanced the regeneration of RGC subtypes that would either die after injury or be resistant to regeneration after *Pten* knockdown. Thus, the choice between *Sox11*-induced axon regeneration or *Sox11-*induced cell death appears to be dependent upon the specific gene expression program of the host cell.

Unexpectedly, we detected an even stronger increase of the *Sox11* short 3′UTR isoform (including CDS) after ONC in animals treated with the AAV-GFP control vector (**Figure 3B**, ONC+GFP), when expression of the long 3′UTR did not change at all. This could be explained with selective AAV and/or GFP toxicity on *Sox11* regulation after ONC. In fact, some studies have reported immunogenic properties for both (Zhu et al., 2009; Ansari et al., 2016; Berns and Muzyczka, 2017), suggesting that the expression of genes related to immune system function is altered in response to AAV delivery. Even though little is known about the role of *Sox11* in immune cells, its overabundance is now well documented in mantle cell lymphoma, a subtype of B-cell lymphomas (Lu et al., 2013). While investigating the relationship between the regulation of *Sox11* mRNA isoforms, optic nerve injury and AAV/GFP toxicity was beyond the scope of our study, future experiments should be designed to investigate this phenomenon. Our finding also reaffirms that care should be taken when analyzing expression results from AAV- and/or GFP-treated samples.

Several non-coding *Bdnf* exons were previously established to be a transcriptional target of SOX11 (Salerno et al., 2012). Thus, we measured expression levels of those *Bdnf* mRNA isoforms known to be regulated by SOX11 to define the tissue-specific expression patterns. First, *Bdnf*-1 was increased 10-fold in DRG, decreased briefly at 7 days, and returned to increased expression at 14 days. This mRNA isoform was not even elevated twofold in the retina. Furthermore, *Bdnf*-4 expression was only minimally upregulated after nerve crush in DRG and not at all in retina. Three other isoforms, *Bdnf*-7, *Bdnf*-8, and *Bdnf*-9a, were expressed at much lower baseline levels and trended toward injury-dependent suppression in DRG but toward an increase in retina. Because the low starting levels of these isoforms, the biological relevance of their fold-changes should be interpreted with caution. *Bdnf*-1 and *Bdnf*-4 were previously found to be the most reactive to depolarization in primary cortical neurons (Pruunsild et al., 2011), and both were reported to be retained in the neuron soma, while other isoforms were found to also be present in axons and dendrites (Chiaruttini et al., 2009). It is interesting to note that the promoters of both mRNA isoforms are repressed selectively at birth (positive H3K27me3, see **Figure 1C**) but that the most likely promoter for *Bdnf-4* is selectively acetylated (H3K27ac) at birth and in adolescence. This demonstrates the tight tissue-specific and spatiotemporal control of *Bdnf* expression. For example, a previous study demonstrated a phenotypic switch in DRG neuron subpopulations expressing BDNF one week after injury from medium-sized, trkA expressing neurons to large-sized, trkB/trkC-expressing neurons (Karchewski et al., 2002). This phenomenon may be the cause of the temporary dip in *Bdnf*-*1* and *Bdnf-4* expression 1 week after sciatic nerve crush as seen in our data, and could be related to the expression of distinct mRNA isoforms.

We observed a decrease in *Bdnf-4* and *Bdnf-1* expression in the retina after ONC and regeneration treatment. This was unexpected, for we anticipated these isoforms to show an increase similar to what we found in the regeneration-prone DRG following sciatic nerve crush (**Figure 2A**). In contrast, in a control group subjected to regeneration treatment but not ONC, no significant changes in expression were found, suggesting that only the combined effect of axon injury and *Pten* inhibition resulted in the suppression of *Bdnf-4* and *Bdnf-1*. This indicates that there is cell type-specific relationship between *Bdnf* mRNA levels and enhanced axon regeneration. PTEN is a known inhibitor of PI3K/AKT signaling, and this pathway is also known to be downstream of BDNF/trkB signaling (Christie et al., 2010). Thus, stimulating the downstream effects of BDNF/trkB signaling combined with injury might induce a negative feedback on *Bdnf* regulation, leading to lower expression levels.

In contrast to acute neuronal injury that can result in chromatolysis with subsequent apoptosis, chronic neurodegeneration is a slow-onset process with a different transcriptional environment (Struebing and Geisert, 2015). We therefore also examined *Sox11* and *Bdnf* mRNA isoform levels in different stages of glaucoma, a blinding disease associated with chronic degeneration of RGCs and optic nerve axons. The only marked change we saw was in the long 3′UTR isoform of *Sox11*, which gradually increased in moderate and severe stages up to twofold. Expression of *Sox11* CDS and short 3′UTR did not change in these aged mice, which was in complete contrast to the ONC+GFP results, where only the CDS and short 3′UTR but not the long 3′UTR showed differences. These results might seem counterintuitive if one assumes that short or long UTR and CDS should be co-expressed in a linear fashion on one RNA strand. However, the findings of expression of distinct RNAs from 3′UTRs or widespread spatial differences in the expression of 3′UTR and CDS of the same gene – which was reported for *Sox11* – may be a challenge to this notion (Mercer et al., 2011; Kocabas et al., 2015). The presence of intragenic histone modifications and CpG islands, as shown for *Sox11* in **Figure 1A**, could be related to this phenomenon. Furthermore, in our analysis of publicly available CAGE data (**Supplementary Figure S1**) we could detect the presence of at least nine mostly intragenic transcription start sites for *Sox11* during ocular development. Thus, *Sox11* 3′UTR and CDS are likely not co-regulated, as those transcripts are either spatially separated, post-transcriptionally modified or selectively degraded. While the biological function of 3′UTR-derived transcripts remains to be determined, based on our findings and those of others, we hypothesize that 3′UTR-derived transcripts could confer a regulatory effect on translation efficiency. In fact, preliminary results from our group suggest that despite a strong increase of *Sox11* mRNA after ONC, the amount of SOX11 protein does not change, and similar processes have been described for other genes (Szostak and Gebauer, 2013).

## Conclusion

We have demonstrated differential expression of *Bdnf* and *Sox11* mRNA isoforms in the PNS and CNS after axon injury. Furthermore, we have shown that *Sox11* expression in the retina is non-linear in regard to its 3′UTR and CDS regions, and that the long 3′UTR and short 3′UTR isoforms are differentially regulated in disease or following experimental intervention. While beyond the scope of the present study, the molecular mechanisms and functional reasons for differential 3′UTR and CDS regulation and how they relate to neuron degeneration and regeneration deserve to be further studied.

## Author Contributions

FS and EG conceived the study in coordination with AE. JW performed all ONC procedures. YL was responsible for intravitreal injections. RK assisted with surgeries and animal breeding and isolated RNA for all samples. OM and AE conducted SCN and DRG isolation. FS performed and analyzed all ddPCR reactions and RACE assays, compiled figures and wrote the paper with input from EG and AE. All authors read and approved the final manuscript.

## Conflict of Interest Statement

The authors declare that the research was conducted in the absence of any commercial or financial relationships that could be construed as a potential conflict of interest.

## Supplementary Material

FIGURE S1 The *Sox11* locus is associated with dynamically regulated, intragenic transcription start sites during ocular development. CAGE data, which capture RNA start sites, were downloaded from the FANTOM database and aligned to the mouse genome (version mm9). There is an upstream antisense TSS during embryonic development stages, and in adulthood, only the canonical TSS is used. TPM, transcripts per million.

FIGURE S2 The expression changes following optic nerve crush for four different microarray probes covering *Sox11* are shown in **(A)**. Data from GeneNetwork (G2 HEI ONC Retina April 2010). Mouse ages ranged from 60 to 90 days. The location of probes relative to the *Sox11* locus are pictured in **(B)**. Higher expression of *Sox11* distal 3′UTR (Probe D) parts are consistent with the ddPCR results from **Figure 2A**. Probes correspond to Illumina Mouse WG6 probe identifiers as follows: ILM610279 (Probe A), ILM106400717 (Probe B), ILM104010731 (Probe C), ILM104920446 (Probe D).

FIGURE S3 Validation of regeneration treatment. Representative retinal flat mounts of animals transfected with either AAV-*Pten*-shRNA or AAV-GFP as control are shown in **(A)**. Staining with an antibody against PTEN demonstrates loss of signal in GFP-positive, successfully transfected retinal ganglion cells only in the AAV-*Pten*-shRNA group. Similarly, only animals having received the regeneration treatment regrow their axons past the optic nerve crush site, marked by an asterisk in **(B)**. Axons were visualized by intravitreal injection of fluorescence-conjugated Cholera toxin B 14 days after ONC. The scale bar represents 20 μm in **(A)** and 100 μm in **(B)**.

FIGURE S4 *Ppia* expression 5 days after optic nerve crush is stable compared to the control situation. This microarray data from genenetwork.org. was created from whole retinas taken from BXD mice, a recombinant inbred mouse cross originating from DBA/2J and C57BL/6J parents.

TABLE S1 Primers used for gene expression analysis were either taken from Salerno et al. (2012) or designed in house.
