# Supplementary figures and images for: Differential Expression of Sox11 and Bdnf mRNA Isoforms in the Injured and Regenerating Nervous Systems
Source: Front Mol Neurosci. 2017 Nov 2;10:354. doi: 10.3389/fnmol.2017.00354 (PMC5701613; doi:10.3389/fnmol.2017.00354)

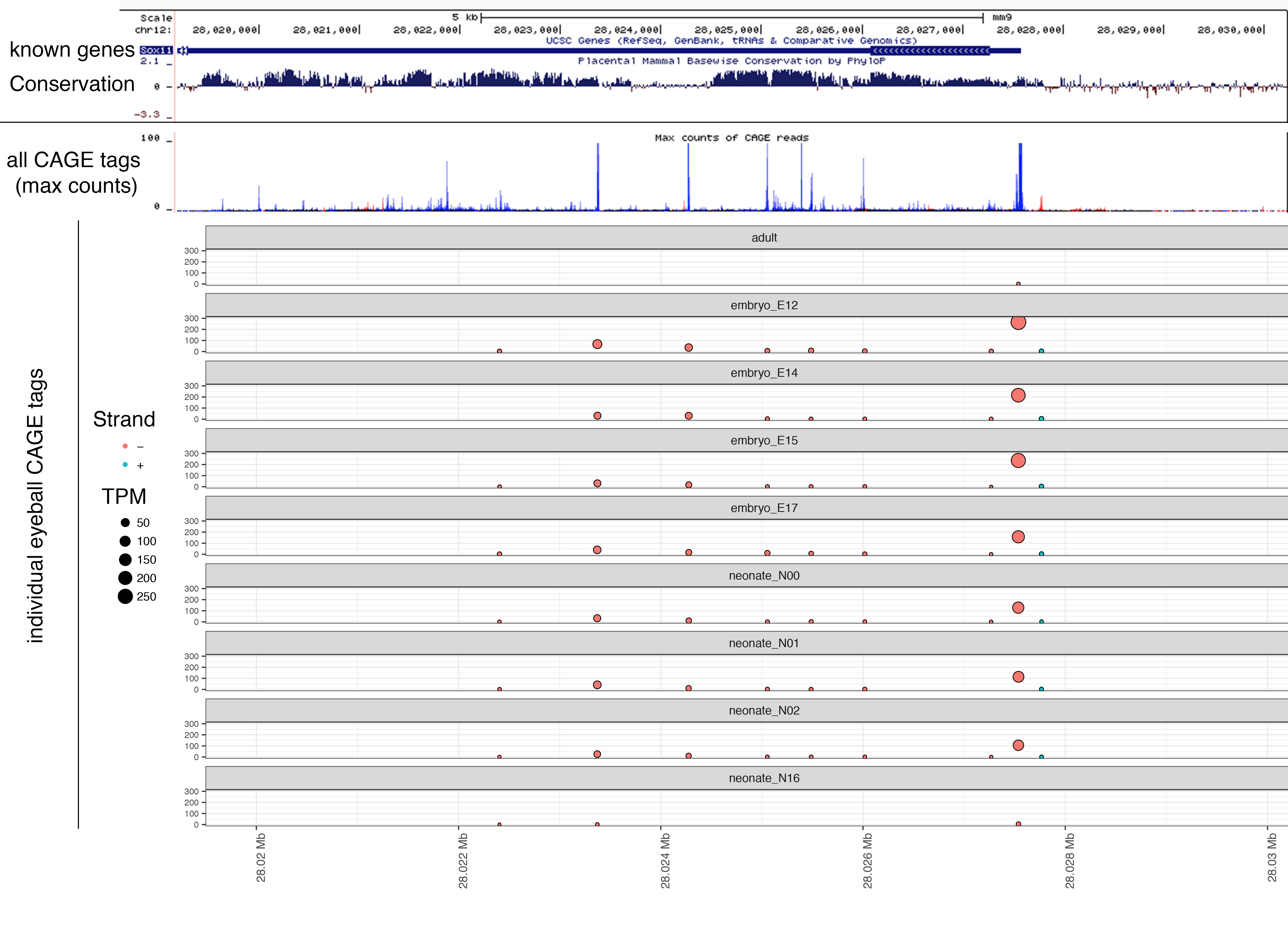

Supplement: FIGURE S1 — The Sox11 locus is associated with dynamically regulated, intragenic transcription start sites during ocular development. CAGE data, which capture RNA start sites, were downloaded from the FANTOM database and aligned to the mouse genome (version mm9). There is an upstream antisense TSS during embryonic development stages, and in adulthood, only the canonical TSS is used. TPM, transcripts per million. [file Image_1.TIF]

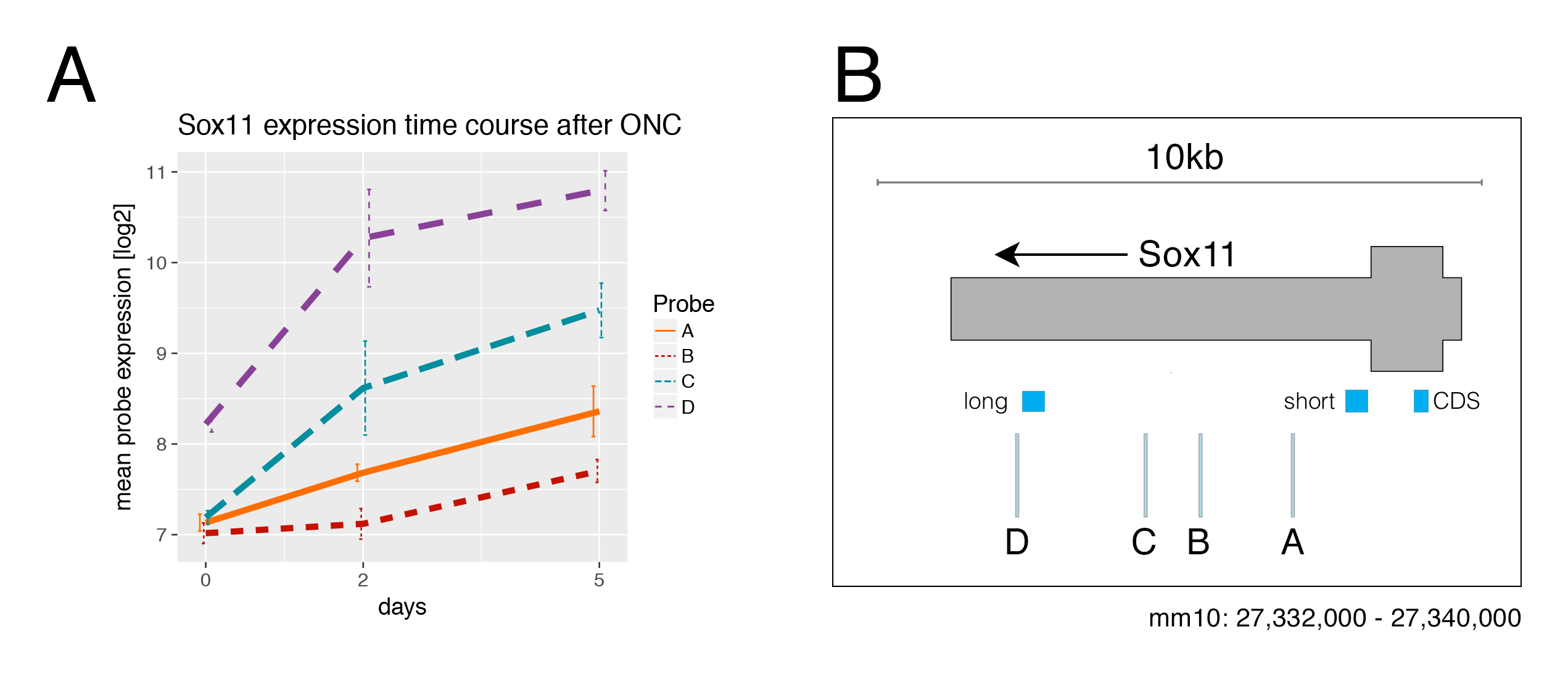

Supplement: FIGURE S2 — The expression changes following optic nerve crush for four different microarray probes covering Sox11 are shown in (A). Data from GeneNetwork (G2 HEI ONC Retina April 2010). Mouse ages ranged from 60 to 90 days. The location of probes relative to the Sox11 locus are pictured in (B). Higher expression of Sox11 distal 3′UTR (Probe D) parts are consistent with the ddPCR results from Figure 2A. Probes correspond to Illumina Mouse WG6 probe identifiers as follows: ILM610279 (Probe A), ILM106400717 (Probe B), ILM104010731 (Probe C), ILM104920446 (Probe D). [file Image_2.TIF]

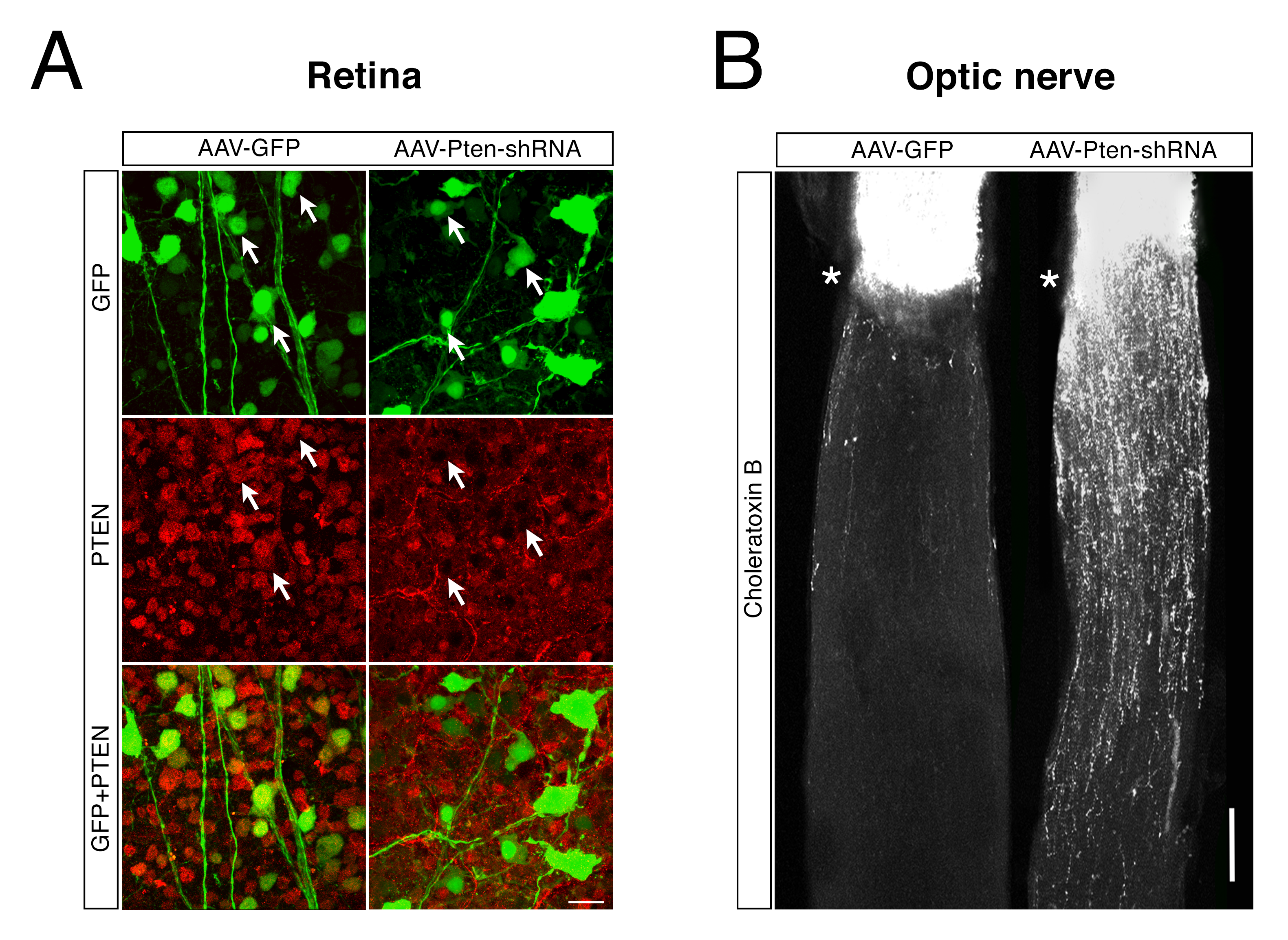

Supplement: FIGURE S3 — Validation of regeneration treatment. Representative retinal flat mounts of animals transfected with either AAV-Pten-shRNA or AAV-GFP as control are shown in (A). Staining with an antibody against PTEN demonstrates loss of signal in GFP-positive, successfully transfected retinal ganglion cells only in the AAV-Pten-shRNA group. Similarly, only animals having received the regeneration treatment regrow their axons past the optic nerve crush site, marked by an asterisk in (B). Axons were visualized by intravitreal injection of fluorescence-conjugated Cholera toxin B 14 days after ONC. The scale bar represents 20 μm in (A) and 100 μm in (B). [file Image_3.TIF]

# Ppia expression after ONC

Control (GN267) vs. ONC 5d (GN385)

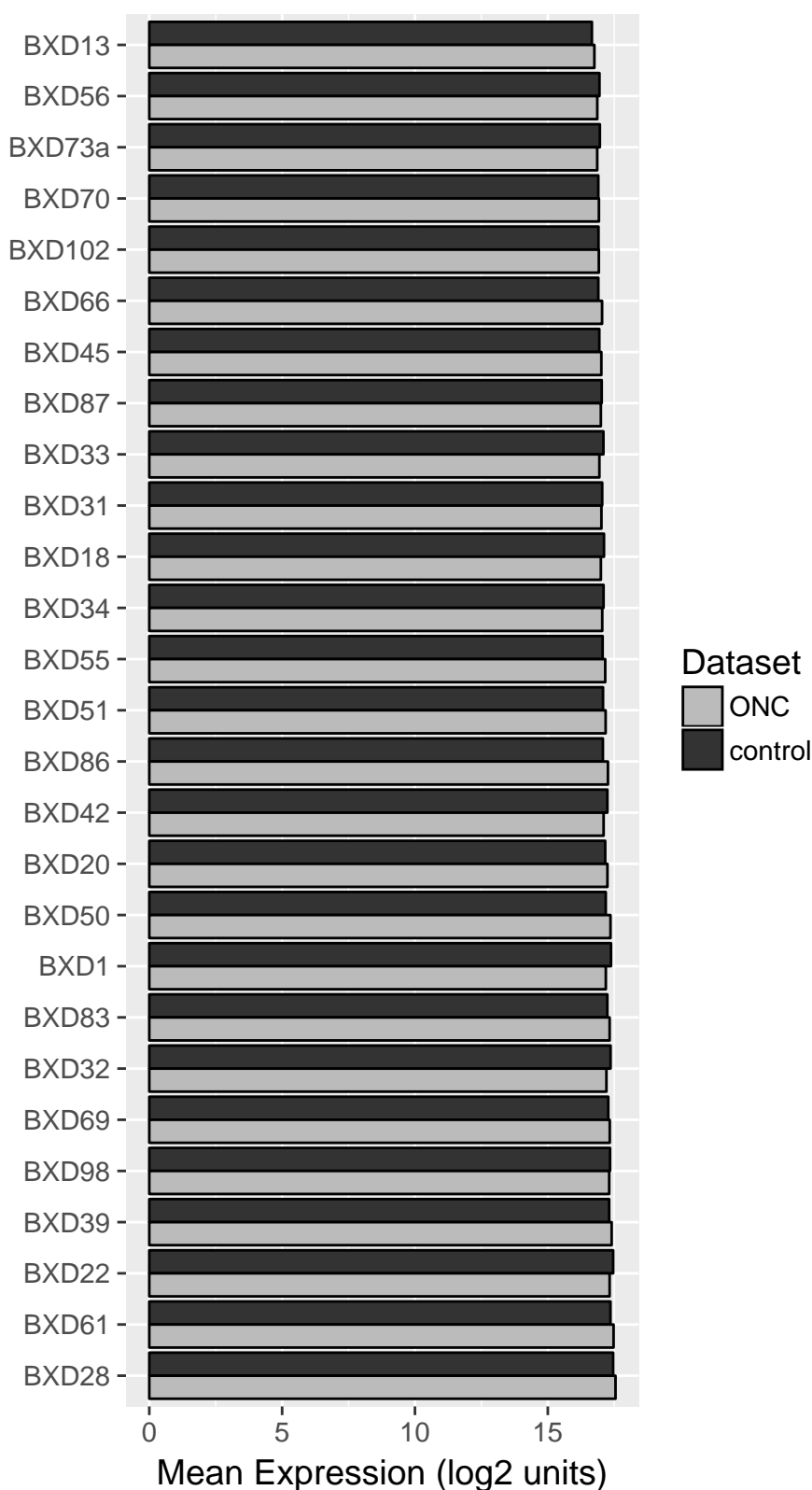

Supplement: FIGURE S4 — Ppia expression 5 days after optic nerve crush is stable compared to the control situation. This microarray data from genenetwork.org. was created from whole retinas taken from BXD mice, a recombinant inbred mouse cross originating from DBA/2J and C57BL/6J parents. [file Image_4.PDF]
